# Model-Based Predictive Control and Reinforcement Learning for Planning Vehicle-Parking Trajectories for Vertical Parking Spaces

**DOI:** 10.3390/s23167124

**Published:** 2023-08-11

**Authors:** Junren Shi, Kexin Li, Changhao Piao, Jun Gao, Lizhi Chen

**Affiliations:** 1 School of Automation, Chongqing University of Posts and Telecommunications, Chongqing 400065, China; s210332009@stu.cqupt.edu.cn (K.L.); piaoch@cqupt.edu.cn (C.P.); chenliz714@gmail.com (L.C.); 2 School of Computer Science and Technology, Chongqing University of Posts and Telecommunications, Chongqing 400065, China; gaoj315@gmail.com

**Keywords:** reinforcement learning, PPO, MPC, autoparking

## Abstract

This paper proposes a vehicle-parking trajectory planning method that addresses the issues of a long trajectory planning time and difficult training convergence during automatic parking. The process involves two stages: finding a parking space and parking planning. The first stage uses model predictive control (MPC) for trajectory tracking from the initial position of the vehicle to the starting point of the parking operation. The second stage employs the proximal policy optimization (PPO) algorithm to transform the parking behavior into a reinforcement learning process. A four-dimensional reward function is set to evaluate the strategy based on a formal reward, guiding the adjustment of neural network parameters and reducing the exploration of invalid actions. Finally, a simulation environment is built for the parking scene, and a network framework is designed. The proposed method is compared with the deep deterministic policy gradient and double-delay deep deterministic policy gradient algorithms in the same scene. Results confirm that the MPC controller accurately performs trajectory-tracking control with minimal steering wheel angle changes and smooth, continuous movement. The PPO-based reinforcement learning method achieves shorter learning times, totaling only 30% and 37.5% of the deep deterministic policy gradient (DDPG) and twin-delayed deep deterministic policy gradient (TD3), and the number of iterations to reach convergence for the PPO algorithm with the introduction of the four-dimensional evaluation metrics is 75% and 68% shorter compared to the DDPG and TD3 algorithms, respectively. This study demonstrates the effectiveness of the proposed method in addressing a slow convergence and long training times in parking trajectory planning, improving parking timeliness.

## 1. Introduction

As an important part of intelligent transportation systems, autonomous driving systems can help or even replace the driver to complete the automatic control of a vehicle and achieve automatic parking. An automatic parking system can complete parking without collisions and without a driver steering wheel, which significantly reduces the probability of parking accidents [1]. Therefore, in recent years, automatic parking systems have become a research hotspot for automotive companies, universities, and R&D units.

The methods used for motion planning in automatic parking can be typically divided into rule-based [2] and learning-based methods [2,3,4]. Compared to these methods, deep reinforcement learning has the advantages of a strong solution capability and the ability to explore autonomously; numerous research scholars and institutions have used deep reinforcement learning to solve control problems [5,6] with excellent results. Although the reinforcement learning algorithm can explore samples autonomously, the contribution of successful samples to the change in neural network weights is easily overburdened because of the random strategy and numerous invalid samples at the early stage of training, resulting in the poor utilization of the samples or even failure to converge [7,8]. Zhang et al. [9] used the artificial-parking control sequence for a fixed position with different heading angles of the starting position of the intelligent body (the terms vehicle, intelligent body, and algorithmic model in this study have the same meaning depending on the context) pretrained to allow the intelligent body to obtain samples with high return values without exploration in the initial stage. Q-Learning, proposed in [10], is an incremental dynamic planning method applied to model-free reinforcement learning in Markov domains. It eventually learns the optimal behavior by continuously evaluating specific behaviors in a given state. However, it requires all behaviors to be repeatedly sampled and the action value function to be discrete. Deep Q-Learning (DQN), proposed in [11,12,13], introduces high-dimensional perception into reinforcement learning through deep learning, combining convolutional neural networks with reinforcement learning. DQN can address discrete, low-dimensional action spaces. However, as the dimensionality of the discrete space actions increases, the number of actions grows exponentially and the curse of dimensionality appears. The large number of actions makes it difficult to achieve an effective search and the discretization process can lead to information loss. Reference [13] proposed deterministic policy gradient (DPG) algorithms to demonstrate that deterministic gradient algorithms are more effective than stochastic gradient algorithms and prove their existence. Deep deterministic policy gradient (DDPG) algorithms are built on DPG using a depth function to approximate and learn the policy such that it can be applied in a high-dimensional, continuous action space [14]. The twin-delayed deep deterministic policy gradient (TD3) algorithm further enhances the learning performance of DDPG by improving the stability and convergence speed of the training process [15]. Bin Zhang [16] stored failed and successful exploration experiences separately and set the sampling ratio that varies with the number of training rounds, so that the intelligent body can always learn from the successful samples. The literature [17] draws inspiration from the Monte Carlo tree search method in AlphaGo [18], generates parking data, and evaluates the data quality with a reward function to filter out low-quality data that may impact the intelligent body during random exploration. Schaul T. [19] takes the TD error as the priority of the samples, and utilizes the SumTree data structure to store the samples, which results in a larger contribution to gradient computation. Priority sampling will help ensure that the samples contributing the most to the gradient computation are selected more easily.

Automatic-parking-path planning algorithms continue to experience certain shortcomings. In the training process, it is still necessary to obtain the samples required for learning using intelligence based on the current strategy interacting with the environment, and the quality of the samples influences the strategy update; the two are interdependent, and the algorithm tends to fall into a local optimum. Compared with robots, cars are incomplete systems [20]. They are laterally and vertically coupled, the parkable space is narrow, and the parking path and control sequence are sparse for a given initial condition. To reduce the learning difficulty, the common means are to fix the starting position training and relax the parking space limit. However, this also leads to the training of the obtained intelligent body compared to the traditional planning method planning ability, which is not strong and cannot meet the practical application of automatic parking requirements.

To further improve the real-time performance of the algorithm, considering that the model predictive control (MPC) method has the feature of being able to meet the demand of multi-constraint control, although it has difficulty guaranteeing an efficient nonlinear system optimization solution operation within the control cycle, this study uses the MPC method to complete the parking-space-finding scenario where lateral control is easy to achieve [21,22,23]. Owing to the use of deep reinforcement learning and data fusion technology, it can accurately predict the vehicle motion state and achieve lateral stability control. Although all the above studies have realized the application of reinforcement learning algorithms in trajectory planning, there remains a major problem in that the output action of the model is discrete, which leads to the nonsmooth motion of the chassis and accelerates damage to the components. To solve this problem, this study applies the PPO algorithm to automatic parking trajectory planning to complete end-to-end reinforcement learning model training [24].

In summary, this paper integrates the MPC method and deep reinforcement learning PPO algorithm into two automatic parking processes by segmenting the parking processes, respectively. Moreover, the PPO algorithm network architecture is improved by considering convergence and stability, and the reward function is designed through four dimensions such that the algorithm can be trained to master environment awareness and safe path planning, and can actively avoid additional dangerous obstacles and find safer paths. Finally, a simulation platform is built to train the intelligent body, analyze and evaluate its performance, and verify the effectiveness of the algorithm.

## 2. Vehicle Model and Environment-Aware Design

### 2.1. Vehicle Kinematic Model

**Figure 1:**
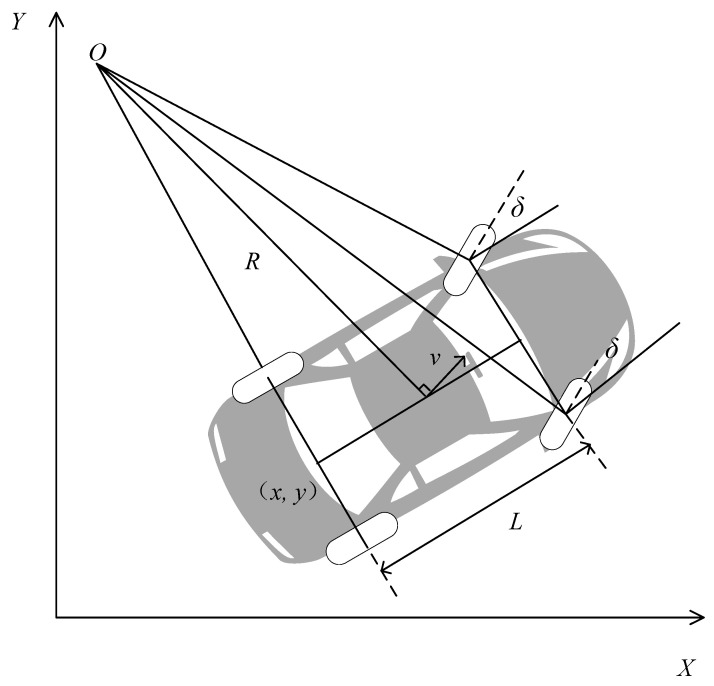
Vehicle kinematic model.

The kinematic constraints in the parking scenario were constructed based on a kinematic model of the entire vehicle. A kinematic model of the vehicle is displayed in Figure 1.

Taking the midpoint of the rear axle of the vehicle as the reference point for the entire vehicle state, the attitude state vector of the entire vehicle is S = [*x, y, φ*]*^T^* and the expression of the kinematic model of the entire vehicle is

(1) $$\left[\begin{array}{c} \dot{x}\left(t\right)\\ \dot{y}\left(t\right)\\ \dot{\varphi }\left(t\right) \end{array}\right]=\left[\begin{array}{c} \cos\left(\varphi \left(t\right)\right)\\ \sin\left(\varphi \left(t\right)\right)\\ \tan\left(\delta \left(t\right)\right)/L \end{array}\right]v\left(t\right),$$

where (*x, y*) are the coordinates of the rear axle midpoint, *φ* is the vehicle heading angle, *L* is the axle distance, *v* is the rear axle speed, and *δ* is the front wheel steering angle.

### 2.2. Environment Perception Design

#### 2.2.1. Self-Position and Velocity Information Perception

**Figure 2:**
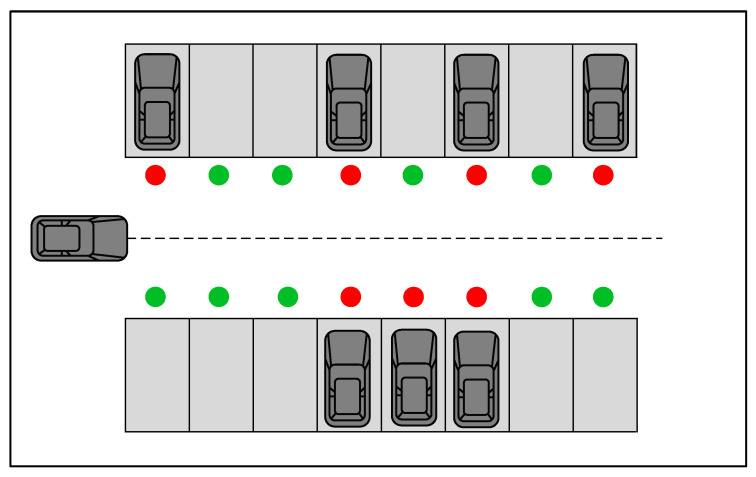
Parking lot mockup.

Common industrial satellite positioning accuracy can only achieve the meter level and cannot meet the requirements of indoor positioning in a small area [25,26]. Therefore, a smart parking lot that can provide a priori information regarding the environment was used as the experimental environment in this study, where the a priori information included parking lot vacancies and vehicle location. As the majority of parking lots consist of rectangles or near-rectangles, the actual scenario could be abstracted into the simulation displayed in Figure 2 based on the symmetry of the parking environment. Based on the geometric properties, we circled the training area within a certain range and transformed the coordinate system based on the upper, lower, left, right, and central parts of the working environment simulation diagram.

**Figure 3:**
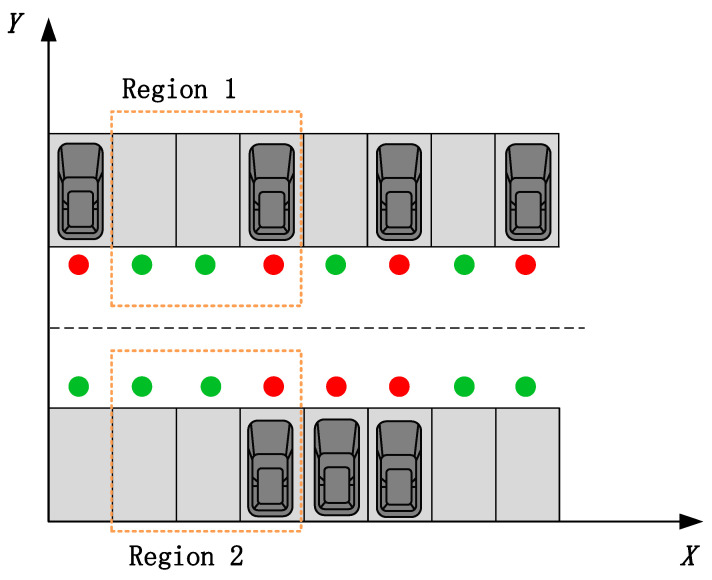
Coordinate transformation.

To make the training more efficient, one side of the parking area was taken for coordinate transformation, thus completing the training of target parking spaces in the entire parking area. As illustrated in the Figure 3 below, take the lower left of the parking area as the origin, and take the horizontal and vertical axes. The horizontal coordinate remains unchanged, while the vertical coordinate increases the distance of the parking space upwards and the direction angle remains unchanged.The green dot means that the parking space is free, and the red dot means that the parking space is occupied. Region 2 can be obtained from region 1 by coordinate transformation.

The coordinate transformation of the vehicle attitude observation from bottom to top at different parking locations was as follows (units: m):

- Part I: $\bar{X}=X,\bar{Y}=Y+20.41,\bar{\theta }=\theta$;
- Part II: $\bar{X}=41-X,\bar{Y}=-64.485-Y,\bar{\theta }=\theta -\pi$;
- Part III: No change;
- Part IV: $\bar{X}=41-X,\bar{Y}=-84.48-Y,\bar{\theta }=\theta -\pi$.

#### 2.2.2. Obstacle Information Perception

**Figure 4:**
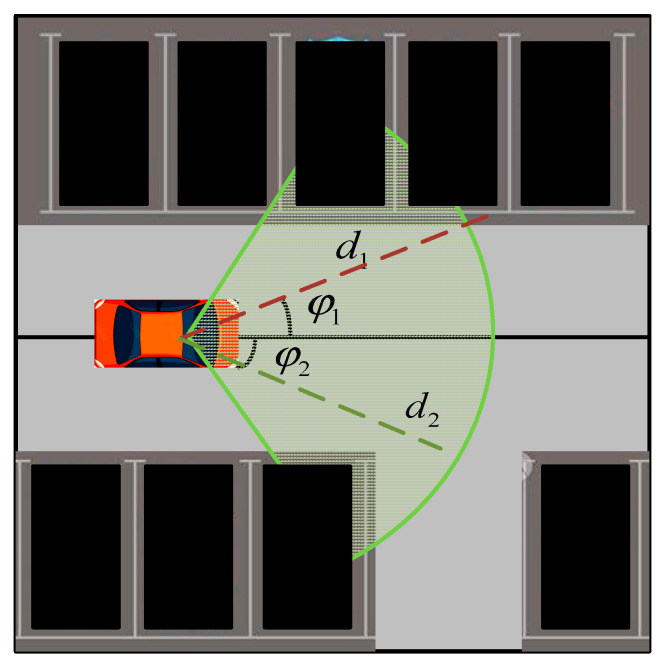
Camera working scene simulation diagram.

For obstacle sensing, a 16-wire LiDAR and camera were selected as obstacle-sensing sensors. The camera module sensing was part of the “finding a parking space” scenario; the vehicle’s own camera provided dynamic information on the parking space in the sensing field of view to determine whether the parking space was vacant or occupied. The camera test range was 120 ° with a depth of 10 m;Its field of view can be seen by the green area in Figure 4. When moving forward from the car, the camera module senses parking spots that fall within the field of view and determines if the parking spot is vacant. This action is achieved using the geometric relationship between the parking spot position and current vehicle pose. A parking spot is within the field of view if *d_i_* ≤ *d_max_* and $\varphi_{min}\leq \varphi_{i}\leq \varphi_{max}$, where *d_i_* is the distance to the parking spot and $\varphi_{i}$ is the angle to the parking spot; the specific coordinates of the parking spot are based on its geometric center.

## 3. Parking Method Framework Design

### 3.1. Overall Framework Design

**Figure 5:**
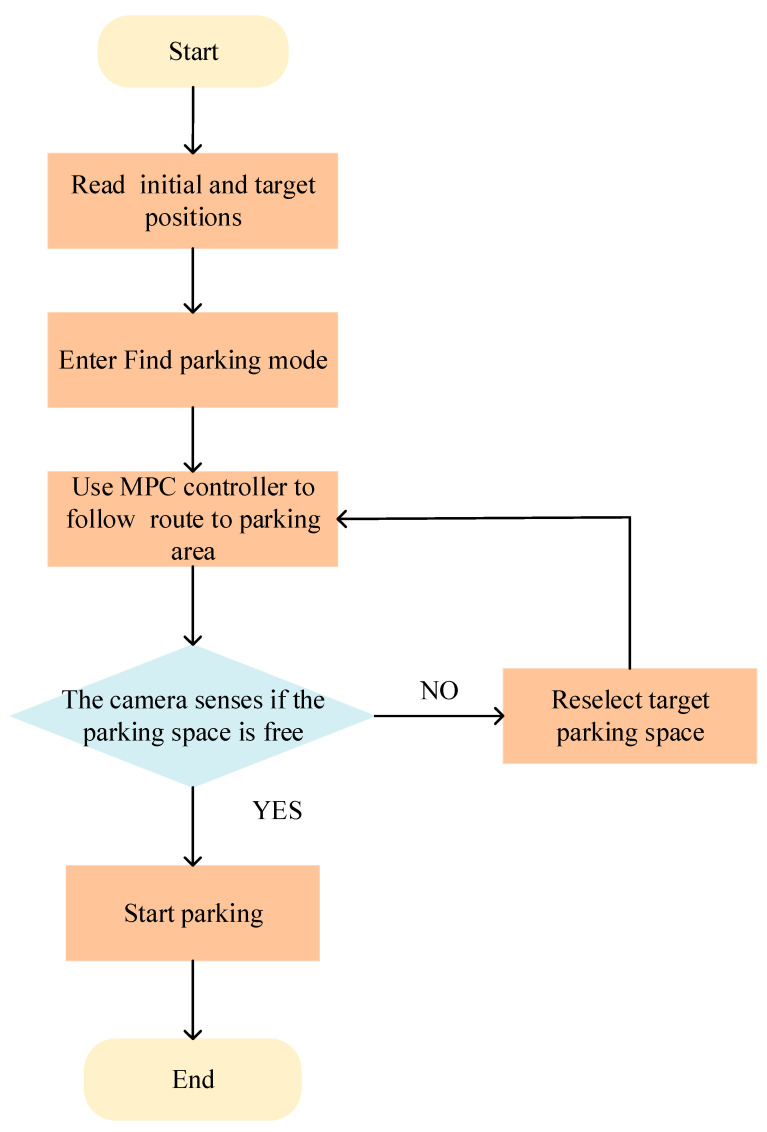
Parking method flow chart.

In this study, the parking process was divided into two scenarios: finding a parking space and parking in a parking space. In the space-finding scenario, the controlled vehicle used the MPC method to track the planned driving trajectory at the vehicle field end. When the parking space was found and the parking action was executed, that is, in the parking space scenario, the controlled vehicle used the proximal policy optimization reinforcement learning algorithm to complete the parking action by controlling the entire vehicle speed and heading angle based on historical experience and the current environment. If the parking error was overly large or there was a parking safety risk, the training mode was opened in the current environment and the trial and error mechanism was used to complete the parking task in this mode, and the experience was updated as shown in Figure 5 below.

In summary, in the case of narrow vertical parking spaces, the MPC and reinforcement learning controllers in this model were placed in the enabled subsystem blocks, which were activated with signals representing whether the vehicle had to search for an empty space or perform a parking action. The enabled signal was determined using the camera algorithm in the vehicle-mode subsystem. Initially, the vehicle was in the search mode and the MPC controller tracked the reference path. When the camera detected that the target parking space was empty, the parking mode was activated, and the reinforcement learning controller performed the parking action.

### 3.2. MPC Controller Design

To meet the demand for real-time online optimal control and improve vehicle stability control performance under multiple constraints, the MPC method was extensively applied to the lateral control of the vehicle. The MPC module controller in this study could obtain a priori information regarding the parking lot environment, including empty parking spaces and its own vehicle position. It combined the parking lot environment information and dynamic influencing factors to control the vehicle and select the fastest route to reach an empty parking space at a uniform speed.

From the above, it can be observed that the vehicle kinematic model can be considered as a control system with the input of the entire vehicle control vector *u* = [*v*, *δ*]*^T^* and the state quantity *x*(*x, y, φ*) for a given reference trajectory, which can be described with the motion trajectory of the reference vehicle, each point on which satisfies the above kinematic equations, where *r* represents the reference quantity in the general form of

(2) $${\dot{\chi }}_{r}=f(\chi_{r},u_{r}),$$

where $\chi_{r}={\left[x_{r}y_{r}\varphi_{r}\right]}^{T},u_{r}={\left[v_{r}\delta_{r}\right]}^{T}$.

The linearized unmanned error model was obtained by applying a Taylor series expansion to Equation (2) at the reference trajectory point, neglecting the high-level term and subtracting it from the general form. To be able to apply the model to the design of the model predictive controller, the results were discretized; the results are represented as Equation (3):

(3) $$\tilde{\chi }(k+1)=A_{k,t}\tilde{\chi }(k)+B_{k,t}\tilde{u}(k),$$

where $A_{k,t}=\left[\begin{array}{ccc} 1 & 0 & -v_{r}\sin\varphi_{r}T\\ 0 & 1 & v_{r}\cos\varphi_{r}T\\ 0 & 0 & 1 \end{array}\right]$, $B_{k,t}=\left[\begin{array}{cc} \cos\varphi_{r}T & 0\\ \sin\varphi_{r}T & 0\\ \frac{\tan\delta_{r}T}{l} & \frac{v_{r}T}{l\cos^{2}\delta_{r}} \end{array}\right]$, and *T* is the sampling period.

In the objective function, the solved variables were the control increments in the control time domain; the constraints could only be in the form of control increments or control increments multiplied by the transformation matrix. Therefore, the objective function was transformed into a standard quadratic form and combined with the constraints, as indicated in Equation (4):

$$J(\xi (t),u(t-1),\Delta U(t))={\left[\Delta U{\left(t\right)}^{T},\varepsilon \right]}^{T}H_{t}\left[\Delta U{\left(t\right)}^{T},\varepsilon \right]+G_{t}\left[\Delta U{\left(t\right)}^{T},\varepsilon \right]$$

(4) $$\begin{array}{l} s.t.\;\Delta U_{\min}\leq \Delta U_{t}\leq \Delta U_{\max}\\ \;\;U_{\min}\leq A\Delta U_{t}+U_{t}\leq U_{\max} \end{array}$$

where $H_{t}=\left[\begin{array}{cc} \Theta_{t}^{T}Q\Theta_{t}+R & 0\\ 0 & \rho \end{array}\right]$, $G_{t}=\left[2e_{t}^{T}Q\Theta_{t}\;0\right]$, and $e_{t}$ is the tracking error in the predicted time domain.

In each control cycle, to complete the optimal solution of the objective function, we obtained the control input increment in the control time domain and the first element as the actual control input increment to act on the system into the next control cycle, and repeated the above process to achieve the tracking control of the vehicle trajectory.

### 3.3. PPO-Based Parking Trajectory Planning Process

#### 3.3.1. Reinforcing the Learning Process

With the rise of artificial intelligence, reinforcement learning algorithms are increasingly used in the field of driverless vehicle control. The basic idea is to gain rewards by interacting with the environment and learning by doing so.

The reinforcement learning algorithm consists of several important parts: the intelligent body, environment, state, action, and reward. The learner and decision maker in reinforcement learning are called agents; the remaining parts that interact with the agent are called the environment. In the reinforcement learning process, the agent observes state parameter *S_t_* in the environment at each time node *t* and makes a behavioral decision *A_t_*. When the agent makes a behavioral decision, the state of the environment moves to the next state *S_t+_*_1_. Moreover, the environment returns to *R_t+_*_1_ based on the behavioral decision *A_t_* makes with the agent in state *S_t_*. The trajectory sequence of the interaction between the intelligence and environment can be expressed as follows:

$$\left\{S_{0},\;A_{0},\;R_{1},\;S_{1},\;A_{1},\;R_{2},\;\ldots ,\;S_{t},\;A_{t},\;R_{t+1}\right\}$$

**Figure 6:**
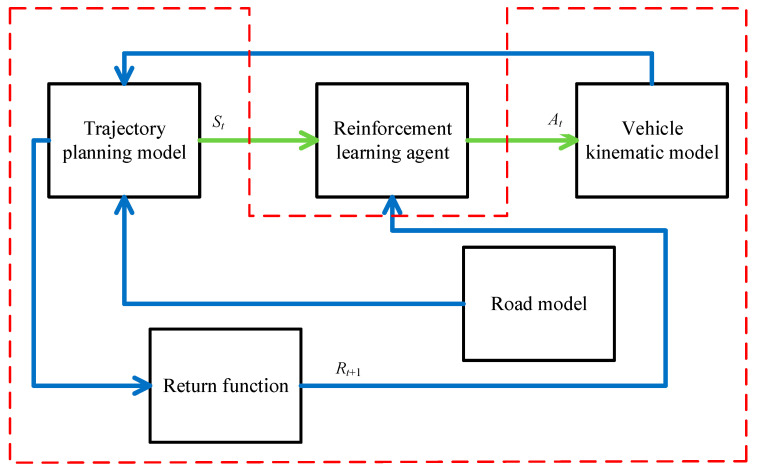
Reinforcing learning process.

As shown in Figure 6 below, reinforcement learning intelligence is unknown to the series of state environment variables during the control and learning process; it learns to update its strategy only with the return value *R_t+_*_1_ and judges whether it exhibits good or bad behavior. Therefore, this study describes the interaction process between the vehicle model and parking space as a trial-and-error reinforcement learning process. In this study, we propose an intelligent vehicle trajectory planning method based on reinforcement learning PPO, which determines the steering wheel angle when tracking the vehicle by observing the lateral deviation, angular deviation, and path curvature between the intelligent vehicle and parking space. We also propose a multi-model fusion method for vehicle trajectory planning that integrates the vehicle model, road model, and return function into the reinforcement learning environment and interacts with the reinforcement learning subject. The main body of reinforcement learning is based on the actor–critic framework; it constructs the corresponding neural network and updates it with the PPO algorithm to complete the learning of the desired steering wheel angle in the process of tracking the trajectory.

#### 3.3.2. PPO Algorithm

In automatic parking trajectory planning for intelligent vehicles, the reinforcement learning subject agent updates its policy using the PPO algorithm (Algorithm 1), which is based on the policy optimization gradient. It is based on the actor–critic framework and can be applied to a continuous behavior space. PPO aims to make the policy select actions with a greater “advantage”, that is, with a considerably greater cumulative reward than predicted by the evaluator. However, we do not want to update the strategies excessively at once, which would likely cause optimization problems. In fact, if a strategy has high entropy, additional rewards are provided to motivate further exploration.
**Algorithm 1:** **PPO algorithm to update neural network process**1. Initialize the weight parameters of the policy network (actor) and value function network (critic). Given the discount factor γ and greedy factor g, the initial state of the vehicle is $\left(x,\;y,\;\theta \right)$.2. Do the following for each time step:●In state $S_{t}$, act $A_{t}$ is selected based on the strategy of joining the noise process to the next state $S_{t+1}$ and receiving the return value $R_{t+1}$;●Save the sequences **(**$S_{t},A_{t},R_{t+1},S_{t+1}$**)** in the recall buffer and randomly sample *n* sets of sequences **(**$S_{i},A_{i},R_{i+1},S_{i+1}$**)**;●Calculate the current critic network value function;●The policy and value functions are made to approximate their true values based on the current state, action, and value function. Among them, the weight parameters of the actor and critic are updated by minimizing the loss $L_{l}$:
  $L_{l}=\frac{1}{n}\sum_{i}\left(y_{i}-Q{\left(S_{i},A_{i}|\theta^{Q}\right)}^{2}\right)$
.
3. Repeat Step 2 until convergence or the maximum number of iterations is reached.

## 4. Trajectory Planning Model Design

### 4.1. Action Strategy Actor–Critic Function Design

The actor–critic network structure is a strategy optimization method commonly used in reinforcement learning. It combines a policy network (i.e., a network that selects actions based on the current state) and a value function network (i.e., a network that estimates the value function of the current state), together with a value function network that is trained simultaneously. During training, the actor network selects actions using the current policy and updates them by sampling the rewards obtained to increase the overall reward. The critic network estimates the value function of its long-term reward based on the current state and assists the actor in updating the policy network such that the sampled rewards are more in line with the desired value function. Actor–critic networks such as continuous control and robot control are widely used in different reinforcement learning tasks.

In this section, the reinforcement learning agent training is performed using the proximal policy optimization algorithm actor–critic architecture, the goal of which is to learn policies that maximize the expected value of the cumulative payoff. To facilitate solving for the optimal policy, the merits of a state and action are evaluated using a value function, which corresponds to the actor network, and a strategy function, which corresponds to the critic network; the output of the actor network is the probability of taking each possible steering action when the vehicle is in a particular state; the output of the critic network is the state value function for that particular stance. In this case, the value function is defined as

(5) $$V(s)=E[\sum_{k=0}^{\infty }\gamma^{k}r_{t+k+1}|s_{t}=s]=E[r_{t+1}+\gamma V(s_{t+1})|s_{t}=s].$$

The policy function is defined as

(6) $$Q(s,a)=E[\sum_{k=0}^{\infty }\gamma^{k}r_{t+k+1}|s_{t}=s,a_{t}=a],$$

where *γ* is the initial learning rate, *r_t+k+_*_1_ is the return at state *s* when the *k*th training at moment *t* takes action *a*, *s_t_* = *s* is the state value at moment *t*, *s_t_* is for state *s*, *s* ∈ {*s*_1_, *s*_2_, …} is for the set of all states, *a_t_* = *a* is the action value at moment *t*, *a_t_* is for action *a*, *a* ∈ {*a*_1_, *a*_2_, …} is for the set of all actions, and *V*(*s_t_*_+1_) is the value function with state *s*_(*t*+1)_ and the cumulative return at state *s_t_*:

(7) $$R_{t}=\gamma r_{t+1}+\gamma^{2}r_{t+2}+\ldots =\sum_{k=0}^{\infty }\gamma^{k}r_{t+k+1}.$$

The state and action value functions respond to the average expected return value of rounds obtainable from the current state or action, and thus can be used as decision indicators for reinforcement learning.

### 4.2. Algorithmic Network Framework Design

The PPO is a strategy optimization method that approximates the solution of the KL dispersion and avoids the regulation of different hyperparameters while ensuring convergence and stability. Based on the above strategy definition, the network architecture of the parking-planning model was designed as follows, including the input layer, actor network, and critic network.

**Figure 7:**
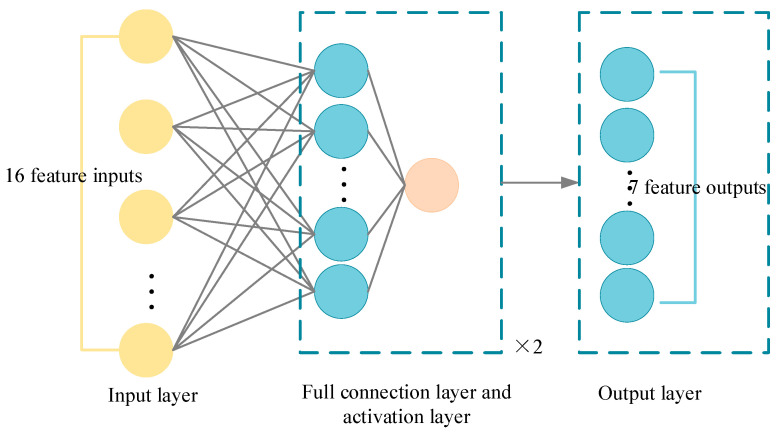
Schematic diagram of actor network framework.

**Figure 8:**
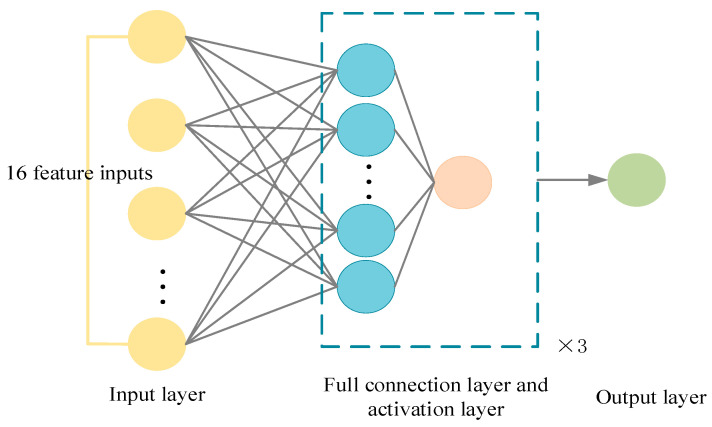
Schematic diagram of critic network framework.

As shown in Figure 7 and Figure 8 below, the input layer is a sequence of state spaces:

### 4.3. Reward Function Design

The reward function is the most critical part of the PPO algorithm and can guide the tuning direction of the deep neural network parameters [27]. According to the principles of reward value design, they can be classified as sparse, formal, or distributed reward functions. In the field of reinforcement learning, the sparse reward function is one of the most common, where the intelligence returns a positive value when it completes the task and zero when it does not [28,29,30,31,32]; however, such a reward function is only suitable for solving single-action problems. For complex environments, the time required to complete the task is overly long, and the design of the reward function based on the above-mentioned reward function is prone to sparse reward functions and the invalidation of gradient information, which is not conducive to the learning of the algorithm. 

Distributed reward functions originate from the probability theory; it is common practice to design reward functions based on a Gaussian distribution [33,34,35,36], which is currently less used. The formal reward function is a reward function that can be applied to a variety of complex environments, where the closer the intelligence is to the target, the larger the reward value obtained is, unlike the sparse reward function. In each state, the intelligent body can obtain the reward value; therefore, the formal reward function is more capable of improving the learning efficiency. According to the above analysis, this study used a formal reward function to design the reward function.

The weight–reward function was designed from the perspective of obstacle avoidance and guidance by considering the relative direction and position of the vehicle, obstacles, and target points in the parking process. In addition, the trajectory reward function was added to the weight–reward function, and a collision-free parking test was conducted using an intelligent vehicle with a combined navigation system for target trajectory acquisition. The powerful learning ability of the PPO algorithm was used for trajectory learning, thus accelerating the convergence of the algorithm and using its reward mechanism to maintain the update amplitude of the control strategy within a reasonable range, reducing the exploration of invalid actions and improving the parking success rate.

For the parking task, the only goal of the intelligent body is to move to the target position, at which time a larger reward should be given to the intelligent body to prevent it from being overshadowed by other partial rewards. To ensure the accuracy of parking, parking is considered complete only when the vehicle moves to the target area and the final positional reward $R_{target}$ is obtained, including the base reward $R_{base}$ and the heading-angle deviation reward.

(8) $$\left\{ \begin{array}{l} R_{target}=R_{base}-\left|\theta -\theta_{target}\right|\\ \left|x_{t}-x_{target}\right|<\varepsilon ,y_{t}<y_{target} \end{array}\right.$$

To ensure safe parking and avoid collisions with other obstacles, a collision penalty, $R_{collision}$, is required. A small yet positive reward value, $R_{c\max}$, is given to the intelligent body when there is no collision to encourage it to explore the unknown area. When the minimum distance *d* between the body and boundary is less than the safe distance $d_{safe}$, a corresponding penalty is applied.

(9) $$R_{collision}=\min\left(R_{c\max},d-d_{safe}\right)$$

In numerous simple tasks (e.g., OpenAI inverted pendulum and mountain bike tasks), with only the above-mentioned mainline reward and collision penalty (sparse reward), intelligence can discover the goal of the task in sufficient explorations. However, parking-path planning is inherently a difficult exploration problem, and even if it is randomly explored one million times, it could possibly not once successfully reach the endpoint; a sparse reward is clearly not applicable. To ensure that the intelligent body receives feedback (dense reward) at all times and guides the intelligent body to move towards the target location, the guidance reward is increased. Distance-based guidance rewards can be considered as follows:

(10) $$\left\{ \begin{array}{l} R_{guide1}=-kd\\ R_{guide2}=k/d \end{array}\right..$$

With the two distance-based rewards described above, despite scaling by factor *k*, the difference between the rewards at longer distances and closer distances can be overly large, causing the intelligence to be excessively “greedy” (spinning in place to gain more) or “reckless” (choosing to collide with obstacles to end the turn so as to not continue receiving negative rewards). Intelligence requires a smoother guidance reward; hence, the guidance reward${\;R}_{guide}$ is set to be calculated from the angle between the center of the vehicle’s rear axle and line connecting the target position and heading angle, with a positive reward for any angle; the smaller the angle (the faster the vehicle approaches the target position), the greater the reward. This enables the intelligent body to learn to not only approach the target position but also think in the long term and avoid choosing a longer detour (to avoid a collision) to reach the reward of the target.

(11) $$R_{guide}=\pi -\left|\arctan\left(\frac{y_{t}-y_{target}}{x_{t}-x_{target}}\right)-\arctan(\theta )\right|.$$

In the reinforcement learning phase, the reward function has a major role in optimizing intelligent-body movements. The reward function was designed to satisfy the safety and stability requirements during the vehicle-parking process. The objective reward function *r_t_* is denoted as

(12) $$r_{t}=2e^{-\left(0.05X_{e}^{2}+0.04Y_{e}^{2}\right)}+0.5e^{-40\theta_{e}^{2}}-0.05\delta^{2}+100f_{t}-50g_{t},$$

where $\left(X_{e},Y_{e},\theta_{e}\right)$ is the error between the position of the self-vehicle and target pose $\left(X_{e},Y_{e}\right)$ and heading angle $\theta_{e}$; δ is the steering angle; $f_{t}$ indicates whether the vehicle has been parked—when it has been parked, its value is “1”, otherwise its value is “0”; and $g_{t}\;$indicates whether the vehicle has collided with an obstacle at time *t*—when a collision has occurred, its value is “1”, otherwise its value is “0”.

When training the intelligent body for trajectory planning, to ensure driving smoothness, the section of each trajectory should not be overly short; based on the length of each trajectory section in vertical parking, the control volume should be minimized to change within a 2 s time [19] and the value of obtaining a penalty should be no less than the guiding role of trajectory reward, while referring to the setting of trajectory reward; as such, the control volume stability indicator reward $R_{flu}$ can be set as

(13) $$R_{flu}=\left\{ \begin{array}{l} -20e^{-(t_{i}-t_{c})}\;\;,\;t_{i}-t_{c}\leq 2\;\\ 0\;\;\;\;,t_{i}-t_{c}>2 \end{array}\right.,$$

where ${\;t}_{i}-t_{c}$ denotes the time difference between the current moment and that of the last change in the control volume.

The final reward function is given with the following equation, where $k_{target}$, $k_{collision}$, $k_{guide}$, and $k_{flu}$ are the reward weights:

(14) $$R=k_{target}R_{target}+k_{collision}R_{collision}+k_{guide}R_{guide}+k_{flu}R_{flu}.$$

Based on the tasks to be accomplished in the vehicle-parking process and the design of the above reward function, it is necessary to define the design principles for the reward function; this study used the weight vector $k=\left[k_{target},k_{collision},k_{guide},k_{flu}\right]$ to design the overall reward function *R*, as in Equation (15).

(15) $$R=\left[k_{target},k_{collision},k_{guide},k_{flu}\right]\left[\begin{array}{l} R_{target}\\ R_{collision}\\ R_{guide}\\ R_{flu} \end{array}\right]$$

The guidance reward function determines the motion direction of the vehicle parking to a certain extent and aims to guide the vehicle into the parking space successfully. If the guidance reward function requires an excessive weight, it can cause the trajectory deviation and corner increment to become larger. Therefore, the guidance reward function in this study uses the smallest weight. In the parking process, the purpose of the obstacle-avoidance reward function designed in this study is to avoid collisions; therefore, the obstacle-avoidance reward function is the reward function that must be used as a priority in the overall reward function; hence, it uses the greatest overall weight. 

The target-reward function designed in this study enables the vehicle to reach the target location faster during the learning process, which is a critical part of the overall reward function. During vehicle operation, no collisions are necessary; therefore, the target-reward function weight is smaller than the obstacle-avoidance reward function weight. 

In summary, the final overall reward function *R* in this study was designed as Equation (16):

(16) $$R=\left[0.25,0.2,0.15,0.4\right]\left[\begin{array}{l} R_{target}\\ R_{collision}\\ R_{guide}\\ R_{flu} \end{array}\right].$$

## 5. Discussion

### 5.1. Simulation Design and Validation

#### Evaluation Index and Scenario Design for Algorithm Training

**Figure 9:**
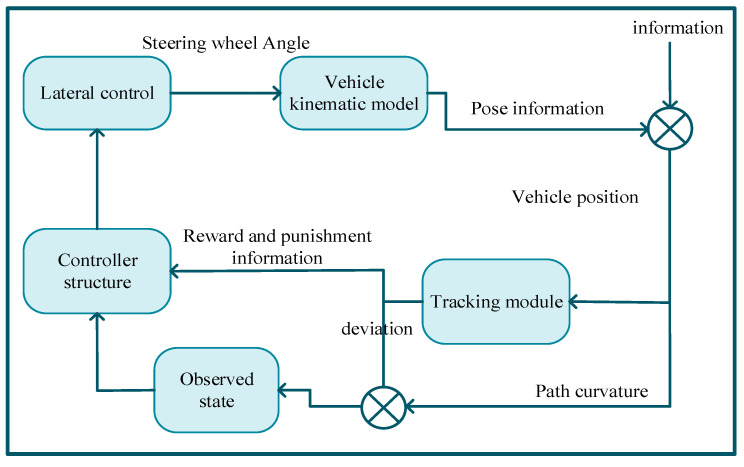
Schematic diagram of simulation framework.

Considering the high cost of training a model directly in a real environment, a simulation environment was built to train the model. The simulation environment in this study was based on a simulator to build a vehicle model and an environment perception model. The effects on the simulation environment after the model was built are displayed in Figure 9.

In this study, a joint MATLAB/Simulink simulation environment is used, and based on the above simulation environment, two types of training scenarios are set up, containing different obstacle distributions and destination areas. When the trajectory-tracking controller finds a free parking space, the trajectory planning controller assumes control and executes the pretrained parking operation. In this part, the intelligent body must preset the conditions. First, because of the symmetry of the parking lot, the lot can be abstracted as a smaller area adjacent to the left and right; here, it is taken as 22.5 m × 20 m, and the target point of parking is its horizontal center. Secondly, the vehicle sensing information is provided by the LiDAR, which uses its radial line segment along the center to determine the proximity of the self-car to other vehicles; the maximum distance is 6 m. Thirdly, the success criterion of the parking behavior is if the error between the center point of the self-car and target center is less than ±0.75 m and ±10^°^. Finally, the parking scene exit mark has three cases: beyond the training range, collision with an obstacle, and successful parking.

In this paper, the interaction process between the vehicle model and the target parking space is described as a trial-and-error reinforcement learning process, which utilizes a depth function to approximate and learn the policy, which is needed to be able to be applied in a high-dimensional, continuous action space. Among the various deep learning methods, the DDPG method can be used for introductory continuous action space DRL algorithms to successfully train working policies on continuous action space tasks. The TD3 algorithm, as an optimized version of the DDPG algorithm, is also a deep reinforcement learning algorithm based on the AC architecture for continuous action space. The above two, as deep reinforcement learning algorithms commonly used for training continuous action space, are used as a control group for the research method and thus to verify the superiority of this research method.

### 5.2. Analysis of Algorithm Training Results

Based on the above training scenarios, the following training strategies were set:

- The number of training rounds was set to 200, and the model parameters were updated every 40 steps; if the round ended, the model was updated and the environment was reinitialized to start the next round of training.
- If the moving platform collided, went beyond the driving range, or reached the destination, the reward was returned, the model parameters were updated, and the initial environment was reinitialized to begin the next round of training.

**Figure 10:**
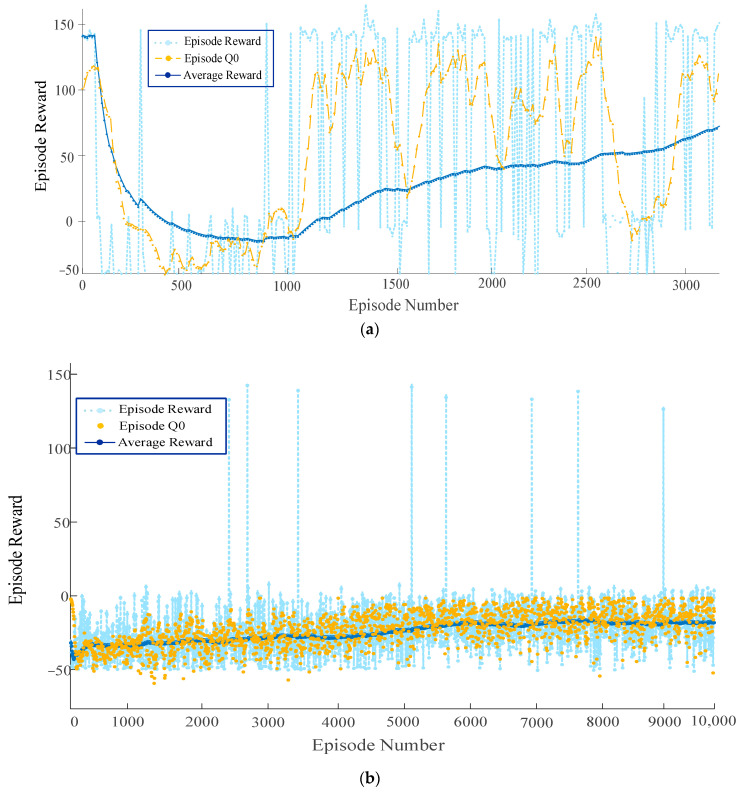

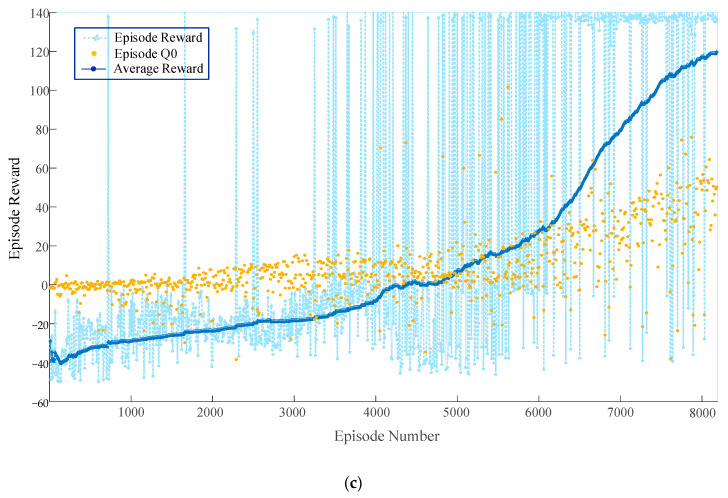
Overall training results. (**a**) Episode reward for rlAutoParkingValet with PPOAgent; (**b**) episode reward for rlAutoParkingValet with DDPGAgent; (**c**) episode reward for rlAutoParkingValet with TD3Agent.

To compare the effect of the proposed algorithm on parking, models based on the proposed algorithm and models based on the TD3 and DDPG algorithms were trained in this study. The training results are displayed in Figure 10.

From the above figure, it can be seen that the training model based on the PPO algorithm successfully parked in the target parking space for the first time in 400 learning sessions, and the first parking success for the DDPG model and the TD3 model were the 2500th and 800th sessions, respectively. Meanwhile, it can be observed that the training models based on the DDPG and TD3 algorithms could not converge after 10,000 rounds of training, whereas the model based on the algorithm proposed in this study achieved convergence after nearly 2500 rounds of training. The training results indicate that the convergence speed of the model based on the proposed algorithm was significantly improved. When the number of training rounds reaches 1000, it can be observed that the fluctuation range of the average reward value of the improved PPO algorithm was significantly better than that of the traditional deep reinforcement learning algorithm, indicating that the effective exploration of the vehicle allows increasingly more empirical data to be obtained under the effect of the improved PPO algorithm. When the number of rounds reached 1500, the average reward value of the improved PPO algorithm indicated a significant increase, whereas the average reward value of the traditional algorithm remained near zero, and there was no significant increase, indicating that the vehicle did not learn a better control strategy. When the number of training rounds reached approximately 2500, the average reward value of the improved algorithm leveled off and remained at approximately 150, indicating that the vehicle had learned the desired control strategy. The convergence and generalization ability of the improved algorithm is studied through examples. Our simulation results indicate that the intelligences trained using the improved algorithms of MPC and PPO have a stronger planning and generalization power as well as larger parkable areas, compared with the traditional deep reinforcement learning methods.

**Figure 11:**
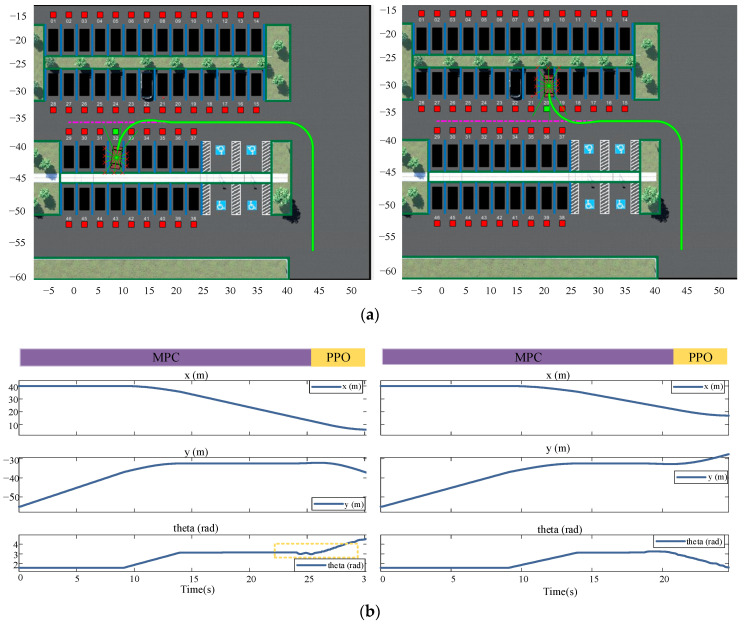
Training-acquired path results and corresponding vehicle position information. (**a**) Parking paths are for spaces No. 32 and No. 20; (**b**) car position information is for parking spaces No. 32 and No. 20.

It should also be noted that, owing to the introduction of the four-dimensional reward function evaluation, the reward was accumulated based on the number of steps; hence, the reward continued to fluctuate after the model converged; however, overall, it did not influence its task of reaching the destination. The model could search for an effective path to a destination and complete the parking task after autonomous learning. The paths learned by the model in the training scenario are displayed in Figure 11.

**Figure 12:**
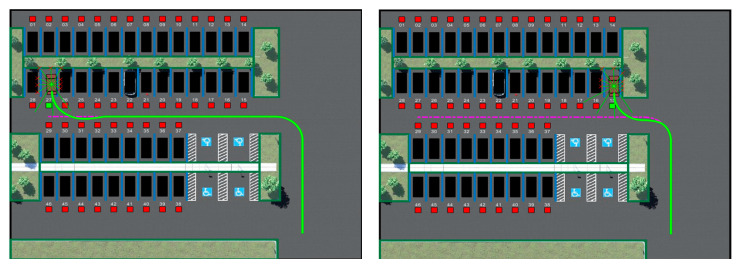
Examples of other target parking trajectories.

Figure 11 displays the trajectory display of the vehicle performing auto-parking when the vehicle is in target-free parking spaces Nos. 32 and 20. The solid green line is the trajectory of the vehicle center coordinates; the coordinate axes of the map are the Cartesian coordinate system used to mark the vehicle position after the abstraction of the parking environment. The coordinate values are displayed in positive and negative coordinates to reduce the training range using the parking lot coordinate conversion. To verify the generality of the parking-planning algorithm obtained from the training in this study, the location of the target parking space in the environment was changed and the starting position was fixed in the lower right corner of the environment. The code was used to control the generation of the target point location; the moving path was obtained as indicated in Figure 11. It can be observed from the figure that the vehicle continued to effectively avoid obstacles after changing the environment. The articulation curve of the two scenes marked by the dashed box in the figure is smooth, with a change amplitude of less than 0.1 *rad* and jitter is not apparent, which means that the vehicle did not have abrupt articulation nodes from the finding-parking-space link to the parking-space link in the entire process, and demonstrated acceptable comfort. Meanwhile, the trajectory planning examples for other target parking spaces in Figure 12 show that this research method can successfully complete parking operations in different environments and has strong generalization.

In summary, this section verified the feasibility of the MPC method using the PPO algorithm for vehicle-parking-path planning. After reconstructing the neural network structure of the PPO algorithm and simulating the parking lot parking environment using simulation software, the MPC and PPO fusion algorithm proposed in this paper and the reinforcement learning algorithm established in the traditional manner were trained in this environment; the results confirm that the method proposed in this paper can make the neural network converge faster and also have a high success rate. The algorithm proposed in this paper can be effectively used in parking-path planning, and its training results have a certain degree of generality, such that when the parking environment changes, the trained neural network can continue to be used for path planning.

## 6. Conclusions

To solve the problems of an excessively long trajectory planning practice and slow training convergence in automatic parking, a parking trajectory planning model based on the PPO algorithm was proposed in this paper for the vehicle automatic parking scenario. Model training and real-world testing were completed with the following main conclusions.

1. The model prediction method was combined with the PPO algorithm to make it more adaptable to parking environments. To solve the problems of traditional trajectory planning algorithms with poor-quality generated paths and sharper points at node connections, this study split the entire parking process into two scenarios: finding a parking space and parking planning, and merged the endpoint of trajectory tracking and the starting point of parking, which effectively improved the smoothness of the paths.
2. A reward function evaluation method based on four-dimensional indicators was designed and a smoothing bias strategy was added such that the intelligent body could learn to approach the target location yet avoid choosing a long detour to reach the reward of the target. This method can substantially accelerate training. The results confirmed that the PPO algorithm with the introduction of four-dimensional evaluation metrics converged in 2500 training cycles, which is 75% and 68% less than the training times of the DDPG and TD3 algorithms, respectively. And the PPO-based reinforcement learning method achieved shorter learning times, totaling only 30% and 37.5% of DDPG and TD3, respectively.
3. To verify the path planning and motion control, a vehicle kinematic model was established based on the Ackermann steering principle and tested in a simulation environment. The test results demonstrated that the model could effectively avoid obstacles and reach the destination under different target positions, thus verifying its effectiveness and acceptable adaptability to the environment. The parking path was smooth without breakpoints, ensuring the comfort of the automatic parking process.

In summary, this study conducted research on automatic parking technology and completed the design and improvement of path planning and motion control algorithms for the characteristics of the parking environment. The fusion of the MPC method and proximal policy optimization algorithm into the automatic parking scenario can significantly improve the planning efficiency of the parking trajectory and generate a smooth route. The results of this study further demonstrate that the method can improve the comprehensive driving performance and timeliness of automatic parking technology in terms of motion planning and control. However, the deep reinforcement learning algorithm designed in this study is applicable only to parking environments without interference. In a daily parking environment, moving objects such as pedestrians and vehicles can cause interference in the parking motion; therefore, subsequent research should focus on the dynamic obstacle-avoidance function.

## Data Availability

The data presented in this study are available on request from the first author.
